# Evolutionary dissection of monkeypox virus: Positive Darwinian selection drives the adaptation of virus–host interaction proteins

**DOI:** 10.3389/fcimb.2022.1083234

**Published:** 2023-01-13

**Authors:** Xiao-Yong Zhan, Gao-Feng Zha, Yulong He

**Affiliations:** The Seventh Affiliated Hospital, Sun Yat-sen University, Shenzhen, China

**Keywords:** monkeypox virus, positive Darwinian selection, virus–host interaction proteins, ankyrin, adaptation, host range

## Abstract

The emerging and ongoing outbreak of human monkeypox (hMPX) in 2022 is a serious global threat. An understanding of the evolution of the monkeypox virus (MPXV) at the single-gene level may provide clues for exploring the unique aspects of the current outbreak: rapidly expanding and sustained human-to-human transmission. For the current investigation, alleles of 156 MPXV coding genes (which account for >95% of the genomic sequence) have been gathered from roughly 1,500 isolates, including those responsible for the previous outbreaks. Using a range of molecular evolution approaches, we demonstrated that intra-species homologous recombination has a negligible effect on MPXV evolution. Despite the fact that the majority of the MPXV genes (64.10%) were subjected to negative selection at the whole gene level, 10 MPXV coding genes (MPXVgp004, 010, 012, 014, 044, 098, 138, 178, 188, and 191) were found to have a total of 15 codons or amino acid sites that are known to evolve under positive Darwinian selection. Except for MPXVgp138, almost all of these genes encode proteins that interact with the host. Of these, five ankyrin proteins (MPXVgp004, 010, 012, 178, and 188) and one Bcl-2-like protein (MPXVgp014) are involved in poxviruses’ host range determination. We discovered that the majority (80%) of positive amino acid substitutions emerged several decades ago, indicating that these sites have been under constant selection pressure and that more adaptable alleles have been circulating in the natural reservoir. This finding was also supported by the minimum spanning networks of the gene alleles. The three positive amino acid substitutions (T/A426V in MPXVgp010, A423D in MPXVgp012, and S105L in MPXVgp191) appeared in 2019 or 2022, indicating that they would be crucial for the virus’ eventual adaptation to humans. Protein modeling suggests that positive amino acid substitutions may affect protein functions in a variety of ways. Further study should focus on revealing the biological effects of positive amino acid substitutions in the genes for viral adaptation to humans, virulence, transmission, and so on. Our study advances knowledge of MPXV’s adaptive mechanism and provides insights for exploring factors that are responsible for the unique aspects of the current outbreak.

## Introduction

The emerging and ongoing outbreak of monkeypox (hMPX) in 2022 has thus far caused more than 82,021 confirmed cases in 110 countries worldwide and is a serious global threat (Centers for Disease Control and Prevention, 2022). As a zoonotic pathogen to humans, the monkeypox virus (MPXV) belongs to the genus *Orthopoxvirus*, which also contains other infectious agents, such as the Vaccinia virus (VACA), the Cowpox virus, and the Smallpox virus (Petersen et al., 2014). In the environment, wild squirrels, wild-living sooty mangabeys, and Gambian giant rats may be natural hosts for MPXV, as indicated by the isolation and the high seroprevalence of the virus in these animals (Radonić et al., 2014; Falendysz et al., 2017), while humans may be the accidental host for MPXV (Farasani, 2022). A recent study speculated on a prevailing hypothesis about the hMPX outbreak in 2022: a single imported case, amplified through one or more super-spreader events because the current 2022 outbreak strains are similar to the strain that caused an outbreak in 2018 to 2019 in Nigeria and traveled to Singapore (Yong et al., 2020; Isidro et al., 2022).

Many studies have shown that the 2022 outbreak of hMPX may be linked to a new lineage, which did not appear before (Isidro et al., 2022; Luna et al., 2022). MPXV has undoubtedly evolved for several decades in its natural hosts, which act as a gene melting pot, unwittingly educating the virus to be more adaptable to humans. With changing epidemiology and increased human-to-human transmission in the 2022 outbreak, it appears that MPXV has become more adaptable to humans, making it a major challenge (Bunge et al., 2022; Kmiec and Kirchhoff, 2022; Velavan and Meyer, 2022; Li et al., 2022). Understanding the evolution of MPXV may therefore provide clues to the key episode of greater adaptation to humans. Wang et al. found that 10 proteins in the MPXV are more prone to mutation, and 24 nonsynonymous mutations were discovered in the 2022 isolates as compared to the 2018 isolates (Wang et al., 2022). A genomic and structural analysis suggests that six mutations in proteins involved in host–pathogen interaction in all MPVX isolates during 2022 may favor viral fitness (Benvenuto et al., 2022). An MPXV mutational study also reveals that the host APOBEC3 plays a role in viral evolution as well as indicators of potential MPXV human adaptation in ongoing microevolution (Isidro et al., 2022). Based on the protein structure study, Kannan et al. indicated that two novel mutations (L108F in F8L and G9R in E4R) could be potential contributing factors to the 2022 outbreak (Kannan et al., 2022). It is unknown whether these MPXV mutations are advantageous traits from the perspective of molecular evolution. Germline or genetic mutations leave behind heritable changes, and natural selection acts on such variations within populations by eliminating deleterious mutations and fixing advantageous ones (Desai and Fisher, 2007; Gregory, 2009). MPXV has about 190 protein-coding genes, which have various functions during the infection (Shchelkunov et al., 2002; Lum et al., 2022). It is necessary to analyze changes in all codons of these genes by comparing MPXV gene alleles from existing isolates to determine the evolutionary impact on the MPXV and reveal the possible advantage of the mutations over the current outbreak isolates. Positive selection, a kind of Darwinian natural selection, is the most crucial evolutionary mechanism (Tiwary, 2022). By using positive selection, the virus could accumulate beneficial mutations to overcome challenging environmental conditions and adapt better to the new host (Asif et al., 2022; Lu et al., 2022). Thus, identifying genes that evolved under positive natural selection is a central goal in studies of molecular evolution for the virus (Venkat et al., 2018). In addition, homologous recombination, also known as intragenic recombination, is another key evolutionary mechanism that can generate genetic variation, which is tested by natural selection, and as such, it also plays an important role in fueling adaptive evolution in the virus (Jouet et al., 2015; Perez-Losada et al., 2015; Tiwary, 2022). To date, over 1,000 MPXV genomes have been sequenced. This can accelerate both genome-wide and single-gene studies on the MPXV. Given that little is known about the evolutionary forces mentioned above acting upon MPXV at the single-gene level, the current study aims to frame the underlying patterns in MPXV evolution at the single-gene level, as well as investigate the current outbreak from an evolutionary standpoint. We incorporate a variety of molecular evolution algorithms to identify MPXV genes that are promoted by intragenic recombination, and, in particular, positive selection. Those codons and MPXV genes that experience positive selection may play key roles in favor of viral survival or human-to-human transmission in the context of the current outbreak, and thus be a potential keystone in the extraordinary 2022 hMPX outbreak. As a result, identifying these genes and codons is crucial for future research. It may provide evolutionary clues for exploring the unique aspects of the current outbreak regarding transmission dynamics, particularly its unprecedented rapid expansion and enhanced and sustained human-to-human transmission.

## Materials and methods

### MPXV strains and study design

About 1,500 sequenced MPXV isolates were enrolled in the study. MPXV genes with lengths more than 300 bp were selected for study since shorter genes lack sufficient variation/alleles for recombination and selection analysis. A total of 156 protein-coding genes were selected for study, accounting for approximately 82% and >95% of MPXV genes in terms of number and nucleotide length, respectively. The details of these genes are shown in **Supplementary Table 1**. The coding genes of MPXV isolate MPXV-USA2003_099_Rope_Squirrel (GenBank accession no. MT903348) were set as references. Nucleic acid sequences of a single gene of MPXV were retrieved by the NCBI Basic Local Alignment Search Tool (BLAST) (https://blast.ncbi.nlm.nih.gov/Blast.cgi) using reference gene sequences as a query. The BLAST program was configured to use Standard databases (nr) of Nucleotide collection (nr/nt) on 25 September 2022, the organism was set as MPXV (taxid:10244), and the algorithm was set as default parameters by increasing the maximum target sequences to 5,000.

### MPXV gene sequence and phylogenetic analysis

The alignment of about 1,500 MPXV isolates downloaded from BLAST was manually checked for integrity. Those truncated gene sequences were removed. Gene sequences were then re-aligned by MEGA X software using Muscle (codons) algorithms (Kumar et al., 2018). Allele profile analyses were performed by using DnaSP 6.12.03 (Rozas et al., 2017). Three to 55 alleles of these genes were obtained (**Supplementary dataset**). The most appropriate nucleotide substitution model for each coding gene was determined by the model finder module of MEGA X and using the Akaike Information Criterion (AIC) (Posada and Buckley, 2004). An unrooted phylogenetic tree of these genes was constructed using MEGA X, with evolutionary history inferred using the Neighbor-Joining (NJ) method and an appropriate nucleotide substitution model obtained by the model finder algorithm (Hasegawa et al., 1985). The percentage of replicate trees in which the associated taxa clustered together in the bootstrap test (1,000 replicates) is shown next to the branches. The evolutionary distances were computed using the Tamura 3-parameter method and are in the units of the number of base substitutions per site (Tamura, 1992). A minimum spanning network (MSN) was constructed by PopART (http://popart.otago.ac.nz) (Bandelt et al., 1999; Leigh and Bryant, 2015), using the alignment of the coding genes to visualize the relationships among gene alleles within MPXV.

### Intra-species homologous recombination analysis

The allele sequences of the protein-coding genes of MPXV were screened by RDP5 to detect intragenic recombination (Martin et al., 2015). Six methods named RDP (Martin and Rybicki, 2000), GENECONV, BookScan (Martin et al., 2005), MaxChi (Smith, 1992), Chimaera (Posada, 2002), and SiScan (Gibbs et al., 2000) that were implemented in the RDP5 were utilized. Recombination was defined as those gene alleles that were found to have recombination events identified by at least three of the methods. All six methods used the same settings: treating sequences as linear, setting statistical significance at *p* < 0.05, and using Bonferroni correction for multiple comparisons. Recombination was also confirmed by the reticulate network tree by observing the side edges in the reticulate network, which commonly arise from recombination, using the SplitsTree5 software (Huson and Bryant, 2006).

### Evolutionary selection analysis

To identify selection pressure operating on genes at the gene or codon level, we used the Maximum Likelihood (ML) method with a visual tool of the codeML software program (Bielawski et al., 2016), named EasyCodeML (Gao et al., 2019). Many codon substitution models were used not only for measuring the average divergence nonsynonymous (dN) and synonymous (dS) ratio of a gene’s substitution, which is commonly used in evolutionary genetic studies and denoted dN/dS, also known as ω, but also for identifying positive selection at the codon level of a gene. First, the topologies of NJ trees for each gene allele were generated by MEGA X, as mentioned above, for the subsequent selection analysis. Multiple seed values were used to fit the model. Codon substitution models include M0 (one ratio, one ω for all sites, indicating an average ω for a whole gene), M1a (nearly neutral, two classes of sites, defined ω0 < 1 or ω1 = 1), M2a (positive selection, allows three site classes including negative, ω0 < 1; neutral, ω1 = 1; and positive, ω2 > 1), M3 (discrete, allows unconstrained discrete distribution of ω among sites), M7 (β, fit to a β distribution for ω among sites), and M8 (β and ω > 1, fit to a β distribution with an extra rate that allows ω = 1). They were typed into null models (M0, M1a, and M7) and positive selection models (M3, M2a, and M8) (Yang et al., 2000). Three nested models, including the M3 vs. M0, the M2a vs. M1a, and the M8 vs. M7 were compared using likelihood ratio tests (LRTs) to assess the best fit of codons. If one of the three nested models showed an LRT *p* < 0.05 (which implies the rejection of null models, also known as the rejection of all codons with ω ≤ 1), the genes were defined as positive selection ones at the codon level. Then, Bayes empirical Bayes (BEB) and Naive Empirical Bayes (NEB) methods were used to identify codons that evolved under positive selection based on a posterior probability of more than 0.90. The average ω values of each gene, which indicate the whole gene selection level (average ω of all codons), were obtained by the M0 model. One exception is MPXVgp059, for which only three alleles in this gene could be found and the ML method could not be used for the calculation (≥4 alleles of the gene are required for using the ML method). Thus, the Nei-Gojobori method implemented in the MEGA X was utilized to estimate the dN/dS of the gene (Luna et al., 2022). Because recombination may impair the ML method’s accuracy in detecting positive selection at the codon level, we used topologies of NJ trees of the gene alleles that exclude the recombinant for positive selection analysis. Fast, Unconstrained Bayesian AppRoximation (FUBAR) (Murrell et al., 2013), a method implemented in the Hyphy package that is based on a Markov chain Monte Carlo (MCMC) routine that ensures robustness against model misspecification by averaging over a large number of predefined site classes, was used to validate the results obtained by the ML method.

### Mapping of positively selected sites to structure models of proteins

The three-dimensional structure of those positive selection genes was modeled using the Phyre server (Kelley et al., 2015). The positive selection sites were mapped onto the structure and visualized by PyMOL (http://www.pymol.org/) (DeLano, 2002).

### Statistical analysis

Continuous variables were compared using the nonparametric Mann–Whitney *U* test. We estimated the frequency rates in each category for categorical variables. The proportions for categorical variables were compared using the chi-square test or Fisher’s exact test (when the data were limited). Statistical significance was defined as *p* < 0.05. GraphPad Prism 8 (GraphPad Software) was used for graphing. Statistical analyses were performed using SPSS 25.0 (IBM software).

## Results

### Intragenic recombination rarely acts on the MPXV

**Figure 1:**
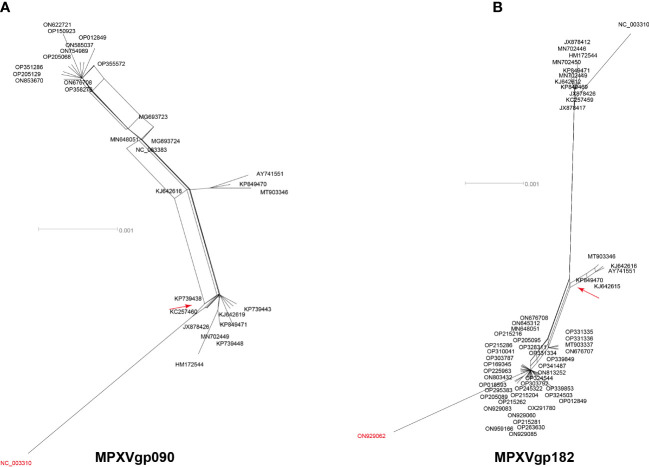
Neighbor-net phylogenetic network shows the relationship among the MPXVgp090 **(A)** and MPXVgp182 **(B)** alleles. The relation between and within alleles is illustrated by splits, representing simultaneously both grouping in the data and evolutionary distances between taxa, highlighting potential conflicting signals or alternative phylogenetic histories (recombination) in the gene molecular evolution. Allele names are shown as their representative MPXV isolate names, shown as GenBank accession numbers, and those that are marked in red indicate that they are recombinants. The network’s internal nodes indicate hypothetical ancestral alleles, while the edges conform to reticulate events like recombination. The red arrow points to a representative reticulate event. Recombination events that were identified by three or more methods were selected and numbered. The parents’ names of the recombinant were indicated according to the RDP5 analysis (see **Table 1**).

Among the 156 analyzed genes, only two genes were found to have undergone a single recombination event during evolution, which generated only one recombinant allele for each gene (**Table 1** and **Figure 1**). These genes were MPXVgp090 and MPXVgp182, which functioned as RNA polymerase subunit and surface glycoprotein of the virus. The discovery of only one recombination event and one recombinant in the two genes suggests that intragenic recombination on the MPXV is rare.

**Table 1:** Intragenic recombination among the gene sequences of monkeypox protein-coding genes by using six different methods implemented in the RDP software.

Recombination events	Recombinant alleles	Major parent^#^	Minor parent^$^	Detection methods implemented in RDP software^^^					
				RDP	GENECONV	Bootscan	Maxchi	Chimaera	SiSscan
MPXVgp090									
1	NC_003310*	N/A	HM172544	N	N^a^	N	Y^b^	Y	Y
MPXVgp182									
1	ON929062	Unknown	AY741551	N	Y	Y	Y	Y	Y

^*^ The allele sequence names are shown as their representative isolates’ genome NCBI accession numbers.

^#^ Major parent: parent allelic sequences contribute to the larger fraction of the sequence.

^$^ Minor parent: parent allelic sequences contribute a smaller fraction of the sequence.

^^^ Recombination events detected by more than two methods are shown.

a N indicates recombination events that were not detected by the selected method.

b Y indicates recombination events that were detected by the selected method. N/A indicates not available.

### Whole gene level negative selection is the main force driving the evolution of MPXV

**Figure 2:**
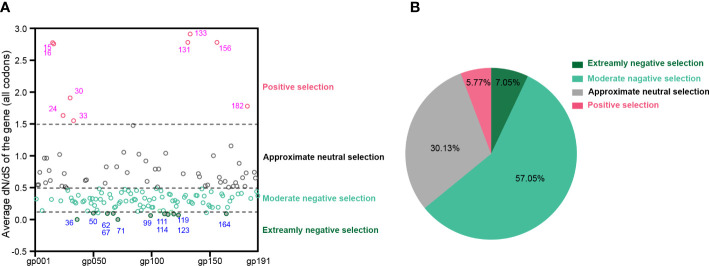
Uniform selective pressure at all sites of an MPXV coding gene. **(A)** The distribution of MPXV genes under different selection pressures. The genes are shown as their protein product names (e.g., MPXVgp001 and MPXVgp002). The blue font shows MPXV genes that have been subjected to extremely negative selection, while the magenta font shows those that have been subjected to positive selection at the whole gene level. Data are shown as dots with median, 25th, and 75th percentile lines. **(B)** The percentage of MPXV genes that are under different levels of uniform selection pressure.

At the whole gene level, most (>94%) MPXV protein-coding genes experienced negative or neutral selection. It should be noted that 11 genes, namely, MPXVgp036, MPXVgp050, MPXVgp062, MPXVgp067, MPXVgp071, MPXVgp099, MPXVgp111, MPXVgp114, MPXVgp119, MPXVgp123, and MPXVgp164, experienced extremely purifying selection with ω < 0.1. Detailed information on these genes is shown in **Supplementary Table 2**. In contrast, nine genes, namely, MPXVgp015, MPXVgp016, MPXVgp024, MPXVgp030, MPXVgp033, MPXVgp131, MPXVgp133, MPXVgp156, and MPXVgp182, experienced positive selection at the whole gene level with an average ω > 1.5 (**Figures 2A, B** and **Supplementary Table 2**). There are 100 genes with ω values less than 0.5, accounting for 64.10% of all, indicating that negative selection is the main force driving the evolution of MPXV (**Figure 2B**).

### Adaptive evolution has a particular impact on genes involved in virus–host interactions, as well as the extraordinary ankyrin genes

**Figure 3:**
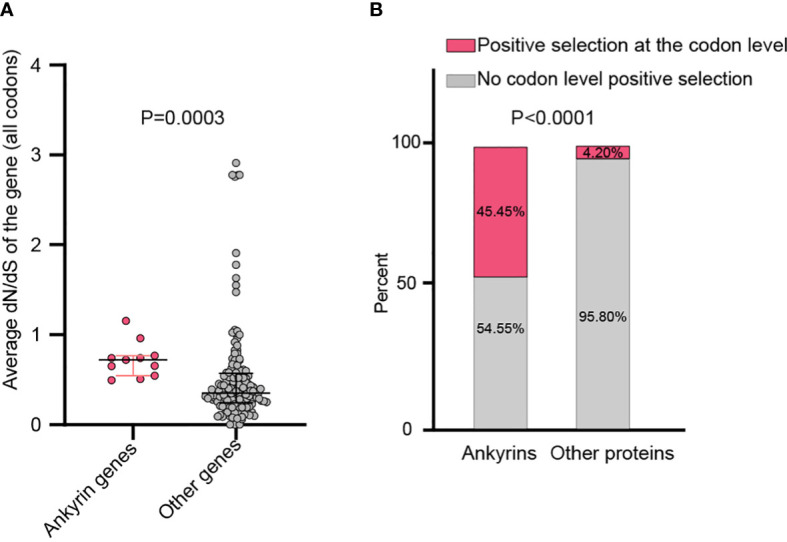
Whole gene and the codon-level positive selection on the ankyrin genes of MPXV. **(A)** Average dN/dS between all the 11 MPXV ankyrin genes and other MPXV genes. Data are shown as dots with 25th, 50th, and 75th percentile lines. The Mann–Whitney *U* test was used for statistical analysis. **(B)** Percentage of ankyrin genes, or other types of genes under positive selection. Chi-square test was used for statistical analysis.

By analyzing all the 156 MPXV genes using three paired LRTs, we found that ten genes, namely, MPXVgp004, MPXVgp010, MPXVgp012, MPXVgp014, MPXVgp044, MPXVgp098, MPXVgp138, MPXVgp178, MPXVgp188, and MPXVgp191, had alternative models (M3, M2a, and M8) that were significantly better fit (*p* < 0.05) than the relevant null models (M0, M1a, and M7), indicating that some codons of these genes were subjected to strong positive selection (**Table 2**). The codon-level positive selection operating these genes was also verified by the FUBAR algorithm, and identical results were obtained (**Table 3** and **Supplementary Table 3**). Detailed information of the 10 genes, including the gene names, lengths, functions, amino acid substitution profiles, and nucleotide mutation profiles of these codons, is shown in **Table 4**. Interestingly, with the exception of MPXVgp098, almost all of these genes encode proteins that interact with the host (Herbert et al., 2015), serve as host cell selection for infection, host immune response regulators, or participate in virion maturation. The most intriguing finding is that half of these genes (MPXVgp004, MPXVgp010, MPXVgp012, MPXVgp178, and MPXVgp188) encode ankyrins, and show more significant positive selection both at the whole gene and at the codon levels (**Figure 3**), despite the fact that only 11 ankyrin genes were found in MPXV genome (based on NCBI genome annotation, detailed information of ankyrin genes is shown in **Supplementary Table 4**). Even more interesting, ankyrins and another virus–host interaction protein, MPXVgp014, a Bcl-2-like protein, have been demonstrated to play roles in determining poxviruses’ host range.

**Table 2:** Log-likelihood values and parameter estimates for the MPXV genes (positive selection ones are shown).

Gene/Model	*nP^*	*lnL*	Estimates of parameters	LRT *p-*value	Positive sites (amino acid sites of the proteins)
MPXVgp004					
M3 (discrete)	33	−1808.429290	*p*0 = 0.94660, *p*1 = 0.00031, ***p*2 = 0.05308**, ω0 = 0.0000, ω1 = 0.00000, **ω2 = 16.10807**	0.0531	4 ***, 239***
M0 (one ratio)	29	−1813.099765	ω0 = 0.74079		Not allowed
M2a (selection)	32	−1808.429325	*p*0 = 0.94967, *p*1 = 0.00146, ***p*2 = 0.05288**, ω0 = 0.0000, ω1 = 1.0000, **ω2 = 16.14577**	**0.0275**	4 ***, 239***
M1a (neutral)	30	−1812.022877	*p*0 = 0.54762, *p*1 = 0.45238 ω0 = 0.0000, ω1 = 1.0000		Not allowed
M8^a^ (beta & ω)	32	−1808.429296	*p*0 = 0.94692, *p* = 0.00500, *q* = 1.3444 ***p*1 = 0.05308, ω=16.10854**	**0.0203**	4 *, 239*
M7 (beta)	30	−1812.328061	*p* = 0.020616, *q* = 0.00783		Not allowed
MPXVgp010&					
M3 (discrete)	37	−2804.209085	*p*0 = 0.90531, *p*1 = 0.00269, ***p*2 = 0.09200**, ω0 = 0.0000, ω1 = 0.0000, **ω2 = 11.83474**	**0.03044**	258 ***, 426 ***, 637 ***
M0 (one ratio)	33	−2809.414812	ω0 = 0.96227		Not allowed
M2a (selection)	36	−2804.209082	*p*0 = 0.90800, *p*1 = 0.0000, ***p*2 = 0.09200**, ω0 = 0.0000, ω1 = 1.0000, **ω2 = 11.83473**	**0.01093**	**258, 426, 637 (all Pr<0.90)**
M1a (neutral)	34	−2808.725356	*p*0 = 0.35210, *p*1 = 0.64790 ω0 = 0.0000, ω1 = 1.0000		Not allowed Not allowed
M8^a^ (beta & ω)	36	−2804.209075	*p*0 = 0.90800, *p* = 0.00500, *q* = 1.09878 ***p*1 = 0.09200, ω=11.83425**	**0.01090**	258*, 426 *, 637*
M7 (beta)	34	−2808.728483	*p* = 0.03361, *q* = 0.01507		Not allowed
MPXVgp012					
M3 (discrete)	47	−2587.302496	*p*0 = 0.01201, *p*1 = 0.98568, ***p*2 = 0.00231**, ω0 = 0.64897, ω1 = 0.65105, **ω2 = 118.65649**	**0.02853**	423 ***
M0 (one ratio)	43	−2592.718210	ω0 = 0.76806		Not allowed
M2a (selection)	46	−2587.302497	*p*0 = 0.99769, *p*1 = 0.0000, ***p*2 = 0.00231**, ω0 = 0.65096, ω1 = 1.00000, **ω2 = 118.62822**	**0.00852**	423 ***
M1a (neutral)	44	−2592.067453	*p*0 = 0.40911, *p*1 = 0.59089 ω0 = 0.0000, ω1 = 1.0000		Not allowed
M8^a^ (beta & ω)	46	−2590.180238	*p*0 = 0.87687, *p* = 0.00500, *q* = 2.27606 ***p*1 = 0.12313, ω=6.59688**	0.1447	423**
M7 (beta)	44	−2592.113143	*p* = 0.01724, *q* = 0.00500		Not allowed
MPXVgp014^@^					
M3 (discrete)	35	−682.492062	*p*0 = 0.00307, *p*1 = 0.97918, ***p*2 = 0.01776**, ω0 = 0.12777, ω1 = 0.12780, **ω2 = 13.16697**	**0.03837**	153 ***
M0 (one ratio)	31	−687.554557	ω0 = 0.31554		Not allowed
M2a (selection)	34	−682.492098	*p*0 = 0.98224, *p*1 = 0.0000, ***p*2 = 0.01776**, ω0 = 0.12780, ω1 = 1.00000, **ω2 = 13.16703**	0.09360	153 ***
M1a (neutral)	32	−684.860852	*p*0 = 0.80994, *p*1 = 0.19006 ω0 = 0.0000, ω1 = 1.0000		Not allowed
M8^a^ (beta & ω)	34	−682.894600	*p*0 = 0.94323, *p* = 0.00500, *q* = 2.67398 ***p*1 = 0.05677, ω=6.36999**	0.08159	153**
M7 (beta)	32	−685.400602	*p* = 0.02169, *q* = 0.06924		Not allowed
MPXVgp044					
M3 (discrete)	43	−2674.58825	*p*0 = 0.0000, *p*1 = 0.99817, ***p*2 = 0.00183**, ω0 = 0.0000, ω1 = 0.49337, **ω2 = 162.92105**	**0.006599**	203***
M0 (one ratio)	39	−2681.702701	ω0 = 0.62132		Not allowed
M2a (selection)	42	−2674.588251	*p*0 = 0.99817 *p*1 = 0.0000, ***p*2 = 0.00183**, ω0 = 0.49325, ω1 = 1.0000, **ω2 = 169.91661**	**0.002770**	203***
M1a (neutral)	40	−2680.477136	*p*0 = 0.55617, *p*1 = 0.44383 ω0 = 0.0000, ω1 = 1.0000		Not allowed
M8^a^ (beta & ω)	42	−2677.568823	*p*0 = 0.93841, *p* = 0.00500, *q* = 2.07484 ***p*1 = 0.06159, ω=11.07608**	**0.04588**	203**
M7 (beta)	40	−2680.650537	*p* = 0.05271, *q* = 0.06315		Not allowed
MPXVgp098					
M3 (discrete)	51	−3498.007392	*p*0 = 0.0000, *p*1 = 0.99640, ***p*2 = 0.00360**, ω0 = 0.0000, ω1 = 0.19605, **ω2 = 52.77541**	**0.02852**	3**, 543**
M0 (one ratio)	47	−3503.423302	ω0 = 0.30333		Not allowed
M2a (selection)	50	−3498.007388	*p*0 = 0.99640, *p*1 = 0.0000, ***p*2 = 0.00360**, ω0 = 0.19607, ω1 = 1.0000, **ω2 = 52.78524**	0.05269	3**, 543**
M1a (neutral)	48	−3500.950791	*p*0 = 0.76240, *p*1 = 0.23760 ω0 = 0.0000, ω1 = 1.0000		Not allowed Not allowed
M8^a^ (beta & ω)	50	−3498.646308	*p*0 = 0.95761, *p* = 0.00500, *q* = 1.69693 ***p*1 = 0.04239, ω=7.54946**	0.0665	3*, 543*
M7 (beta)	48	−3501.356812	*p* = 0.00500, *q* = 0.00694		Not allowed
MPXVgp138					
M3 (discrete)	99	−2270.364472	*p*0 = 0.0000, *p*1 = 0.94970, ***p*2 = 0.05030**, ω0 = 0.0000, ω1 = 0.09662, **ω2 = 6.38119**	**<0.00001**	205 ***
M0 (one ratio)	95	−2285.601424	ω0 = 0.37838		Not allowed
M2a (selection)	98	−2270.365494	*p*0 = 0.94967, *p*1 = 0.00008, ***p*2 = 0.05025**, ω0 = 0.09666, ω1 = 1.0000, **ω2 = 6.38466**	**0.003318**	205 ***
M1a (neutral)	96	−2276.073841	*p*0 = 0.77775, *p*1 = 0.22225 ω0 = 0.0000, ω1 = 1.0000		Not allowed Not allowed
M8^a^ (beta & ω)	98	−2270.377125	*p*0 = 0.94987, *p* = 0.57961, *q* = 4.61694 ***p*1 = 0.05013, ω=6.38213**	**0.00116**	205 ***
M7 (beta)	96	−2277.135432	*p* = 0.00666, *q* = 0.02262		Not allowed
MPXVgp178					
M3 (discrete)	55	−3234.202713	*p*0 = 0.0000, *p*1 = 0.99683, ***p*2 = 0.00317**, ω0 = 0.00000, ω1 = 0.52285, **ω2 = 62.79922**	**0.03911**	689 ***
M0 (one ratio)	51	−3239.242595	ω0 = 1.77937		Not allowed
M2a (selection)	54	−3234.202647	*p*0 = 0.99683, *p*1 = 0.00441, ***p*2 = 0.01728**, ω0 = 0.32017, ω1 = 1.00000, **ω2 = 14.74808**	**0.01757**	689 ***
M1a (neutral)	52	−3238.244122	*p*0 = 0.48293, *p*1 = 0.51707 ω0 = 0.0000, ω1 = 1.0000		Not allowed
M8^a^ (beta & ω)	54	−3235.876765	*p*0 = 0.91074, *p* = 0.00500, *q* = 2.8176 ***p*1 = 0.08926, ω=7.93303**	0.07363	689 **
M7 (beta)	52	−3238.485516	*p* = 0.04221, *q* = 0.03779		Not allowed
MPXVgp188					
M3 (discrete)	33	−1809.049451	*p*0 = 99767, *p*1 = 0.0000, ***p*2 = 0.00233**, ω0 = 0.64737,ω1 = 5.47829, **ω2 = 435.15062**	0.1201	239***
M0 (one ratio)	29	−1812.707933	ω0 = 0.74175		Not allowed
M2a (selection)	32	−1809.049133	*p*0 = 0.99767, *p*1 = 0.0000, ***p*2 = 0.00233**, ω0 = 0.64736, ω1 = 1.00000, **ω2 = 435.07177**	**0.03831**	239***
M1a (neutral)	30	−1812.311139	*p*0 = 0.1842, *p*1 = 0.58158 ω0 = 0.0000, ω1 = 1.0000		Not allowed
M8^a^ (beta & ω)	32	−1810.938155	*p*0 = 0.91350, *p* = 0.00500, *q* = 1.42513 ***p*1 = 0.08650, ω=9.57356**	0.2446	**N/A**
M7 (beta)	30	−1812.346467	*p* = 0.02986, *q* = 0.01303		Not allowed
MPXVgp191^$^					
M3 (discrete)	39	−1100.910355	*p*0 = 0.00305, *p*1 = 0.95835, ***p*2 = 0.03860**, ω0 = 0.0000, ω1 = 0.0000, **ω2 = 12.49552**	**0.01762**	86 ***, 105 ***
M0 (one ratio)	35	−1106.892432	ω0 = 0.37267		Not allowed
M2a (selection)	38	−1100.910163	*p*0 = 0.96140, *p*1 = 0.0000, ***p*2 = 0.03860**, ω0 = 0.000, ω1 = 1.0000, **ω2 = 12.49516**	**0.02696**	86***, 105***
M1a (neutral)	36	−1104.523584	*p*0 = 0.76009, *p*1 = 0.23991 ω0 = 0.0000, ω1 = 1.0000		Not allowed Not allowed
M8^a^ (beta & ω)	38	−1101.255770	*p*0 = 0.96133, *p* = 0.00500, *q* = 2.25829 ***p*1 = 0.03867, ω=12.46335**	**0.04273**	86*, 105*
M7 (beta)	36	−1104.408649	*p* = 0.00705, *q* = 0.01079		Not allowed

^^^p denotes the number of parameters in the ω distribution; lnL denotes the log-likelihood; ω is the ratio of dN/dS, and LRT p-value denotes the value of the chi-square test. Positive selection parameters are shown in bold, where p2 indicates the proportion of positive sites and ω2 indicates average ω of the positive selection sites obtained by the models; positive selection sites were identified using Bayes empirical Bayes (BEB) methods under the M8 model or by Naive Empirical Bayes (NEB) or Empirical Bayes methods under M3 and M2a models.

^&^ Although M2a vs. M1a yields an LRT p < 0.05, the posterior probabilities for the potential positive selection sites 258, 426, and 637 are all less than 0.90.

^@^ The C-terminal of MPXVgp014 varies among different alleles and is shown as “DDDR”, “DDDDE”, or DDDDDDDDR/D, and so on, resulting in a variation in the number of amino acid residues of the protein (153 to 158) among alleles, and the site nomenclature was based on the 2022 outbreak allele ON645312.

^$^ MPXVgp191 has two distinct protein profile groups, one of which encodes an additional six amino acid sequence “EDDEVS” between sites 67 and 68 (e.g., allele MT903348). The position of the proteins’ positive sites is shown here as the position in the 2022 outbreak gene alleles.

The posterior probabilities (Pr) ≥ 0.90, (Pr) ≥ 0.95, and Pr ≥ 0.99 are indicated by *, **, and ***, respectively.

**Table 3:** Parameter estimates for protein-coding genes of monkeypox virus and positive selection sites detected by Fast Unconstrained Bayesian Approximation methods implemented in the HyPhy package.

Genes/positive selection sites	α	β	Bayes factor [β > α]	Posterior Pr [β > α]
MPXVgp004				
4	4.292	33.92	15.721	0.930
239	4.574	34.006	14.538	0.925
MPXVgp010				
258	3.367	32.228	19.401	0.944
426	3.015	31.287	21.225	0.949
637	3.955	35.52	18.474	0.941
MPXVgp012				
423	6.031	36.63	11.016	0.900
MPXVgp014#				
153	4.099	38.139	21.07	0.947
MPXVgp044				
203	3.897	41.5	27.855	0.958
MPXVgp09				
3	3.063	30.57	21.573	0.943
543	3.052	29.582	20.548	0.941
MPXVgp138				
205	3.039	22.398	18.529	0.933
MPXVgp178				
689	3.53	36.696	25.246	0.955
MPXVgp188				
239	4.579	35.345	15.533	0.929
MPXVgp191#				
86	2.272	29.772	27.652	0.959
105	3.402	31.009	18.047	0.939

α indicates (dS) value and β indicates (dN) value.

*Cutoff value for posterior Pr in FUBAR was set at 0.90 based on the research reported by Ben Murrell et al. [Mol. Biol. Evol. 30(5):1196–1205 doi:10.1093/molbev/mst030].

#Due to some gaps that were generated in the process of coding gene alignment, the nomenclature of these sites is corrected to be consistent with the codeML method.

**Table 4:** Description of genes under positive selection at the codon level.

Gene product (based on the annotation of MT903348)	Gene	Gene (VACV nomenclature)	Sequence length (aa)	Substitution profiles (aa) (relative to the 2022 outbreak isolates)	Positive mutation profiles ()	Description	Function	Virus–host protein interactions	Ref.
MPXVgp004	D1L	N/A	437	G4V, A4V; L239W	11G>T, 11C>T; 716T>G	Ankyrin	Host range	Y#	(Herbert et al., 2015)
MPXVgp010	D7L	N/A	659	L258S; A426V, T426V; E637K	773T>C; 1276A>G, 1277C>T; 1909G>A	Ankyrin	Host range	Y	(Spehner et al., 1988; Herbert et al., 2015)
MPXVgp012	D9L	C9L	630	A423D	1268C>A	Ankyrin	Host range	Y	(Herbert et al., 2015)
MPXVgp014*	D11L	C6L	153 to 158	E153R; D153R	457G>A,458A>G; 457G>A, 458A>G, 459T>A	Bcl-2-like protein, IFN-beta inhibitor	Suppression of host immune response, host range	Y	(Gonzalez and Esteban, 2010)
MPXVgp044	C18L	F12L	635	Y203C	608A>G	EEV maturation protein	Virion association, wrapping membrane, interaction with host KLC2	Y	(Laliberte and Moss, 2010)
MPXVgp098	E1R	D1R	845	T3A; T543A	7A>G; 1627A>G	mRNA capping enzyme subunit	Catalyzes the three steps of mRNA capping and regulates gene transcription	N#	(De la Peña et al., 2007)
MPXVgp138	A28L	A26L	507 to 515	H205R	614A>G	Component of IMV surface tubules	Participate in the binding of MVs to the cell surface	Y	(Moss, 2016)
MPXVgp178	B17R	B20R	787 to 789	C689R; H689R	2065T>C; 2066A>G	Ankyrin	Host range	Y	(Herbert et al., 2015)
MPXVgp188	N4R	N/A	437	L239W	716T>G	Ankyrin	Host range	Y	(Herbert et al., 2015)
MPXVgp191$	J3R	C23L	246 to 252	N86T; S105L	257A>C; 314C>T	Chemokine binding protein	Modulate host immune response	Y	(Townsend et al., 2013; Townsend et al., 2017)

* The number of MPXVgp014 amino acid residues was not the same (153 to 158) among different alleles, and the length was calculated using the 2022 outbreak allele ON645312.

^$^ MPXVgp191 has two distinct protein profile groups, one of which encodes an additional six amino acid sequence “EDDEVS” between sites 67 and 68 (e.g., allele MT903348). The position of the proteins’ positive sites and the DNA mutation profiles are shown here as the position in the 2022 outbreak gene alleles.

^#^ Y is the abbreviation of “Yes”, which means the existence of virus–host protein interactions in these proteins, whereas N indicates the opposite.

N/A indicates not available.

### Phylogeny revealing a novel viral clade with positive amino acid substitutions similar to early-emerging isolates

**Figure 4:**
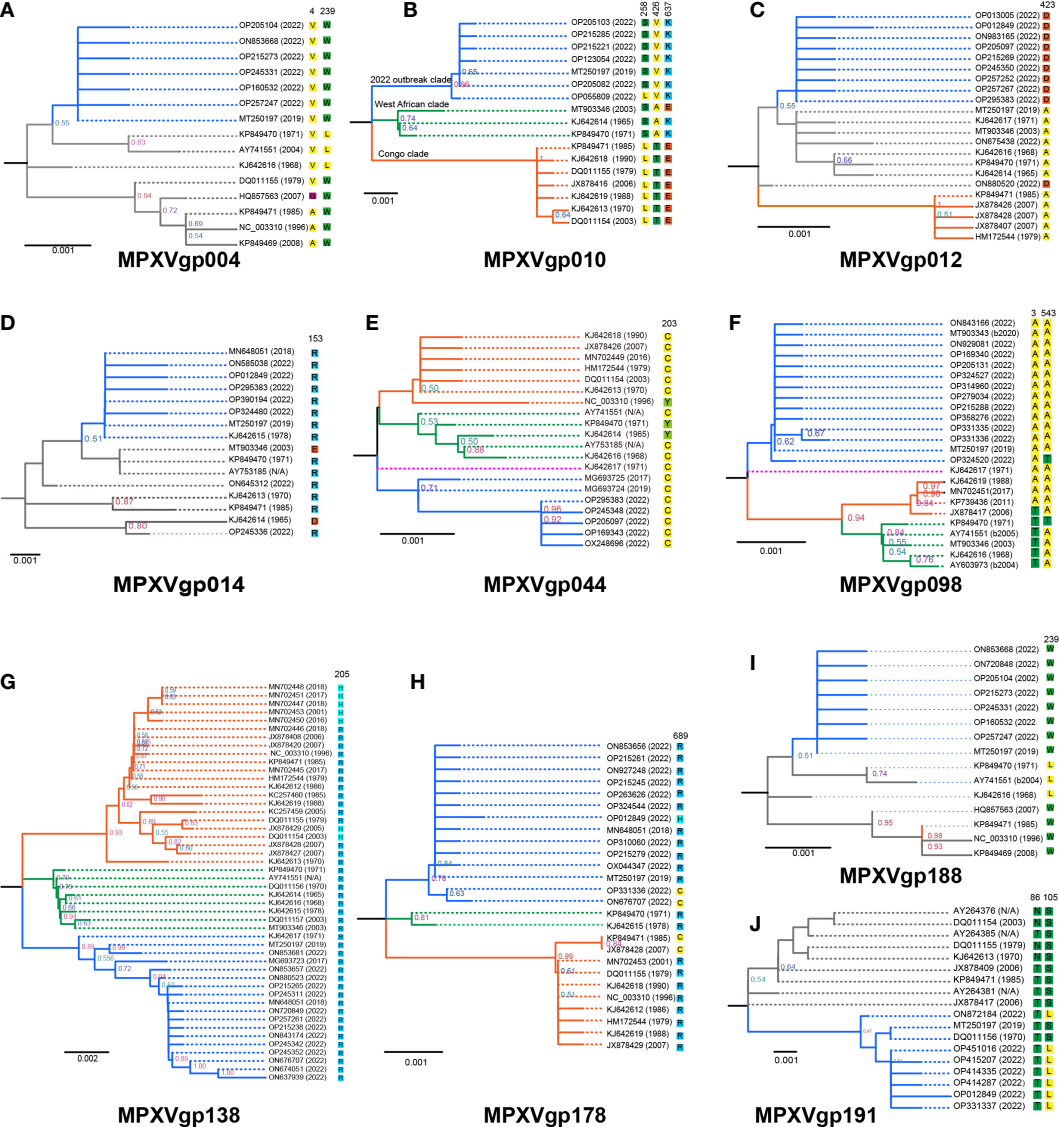
Phylogeny of MPXV genes evolved under positive selection at the codon level and amino acids at the positive selection sites in the 10 MPXV genes. Those genes that were identified as positively selected genes at the codon level include **(A)** MPXVgp004, **(B)** MPXVgp010, **(C)** MPXVgp012, **(D)** MPXVgp014, **(E)** MPXVgp044, **(F)** MPXVgp098, **(G)** MPXVgp138, **(H)** MPXVgp178, **(I)**. MPXVgp188, and **(J)** MPXVgp191. Interior branch numbers represent bootstrap values and are indicated when the values are greater than 0.5. Allele names were marked as their representative isolate names, shown as GenBank accession numbers, and the earliest possible year for the allele to arise is shown. Each allele’s amino acid substitution in positive selection sites is depicted. The isolate ON880520 MPXVgp012 gene has seven nucleotides (overlap amino acid residues 835 to 839) that were not obtained by sequencing and are shown as “N”, and these nucleotides were filled with the most identity nucleotides. Each allele of the 10 genes was re-BLASTed to determine the first time they appeared in an isolate based on the annotation. Branches of the same color are clustered into a clade. The blue clade represents the “2022 outbreak clade”, while the orange and blue represent the “Congo and West African clades” as shown on MPXVgp010 as an example. The purple clade represents a singleton of alleles that make up an additional clade. The gray branches indicate that the branch topology did not allow them to be precisely classified into the three clades mentioned above. N/A denotes that the year the representative isolate arose could not be determined. Based on the analysis of the isolates harboring these alleles, B2020, B2004, and B2005 indicate that these alleles arose no earlier than 2020, 2004, or 2005.

The phylogeny of MPXV genes that underwent codon-level positive selection revealed that the majority of them consisted of three main clades, with those alleles only found in isolates from 2022 being evolutionary distinct from the others (**Figures 4A–J**). This was very obvious in the genes, including MPXVgp010, MPXVgp044, MPXVgp098, MPXVgp138, and MPXVgp178 (**Figures 4B, E–H**), and could be categorized as the 2022 outbreak, the West African, and Congo clades, based on the representative isolate information. In general, we could observe a close relationship between the 2022 isolates and the 2019 (MT250197 and MG693724) or 2017 isolates (MG93725 and MG693723) based on these 10 genes (**Figure 4**). However, some certain unusual situations in the phylogeny of these genes should be highlighted. The MPXVgp012 alleles ON880520 and ON675438, for example, which first appeared in 2022, formed unique clades that differed significantly from the clade formed by 2022 isolates (**Figure 4C**). A similar result was observed on MPXVgp014, with the 2022 allele OP245336 and the 1965 allele KJ642614 forming a distinct clade (**Figure 4D**).

The positive amino acid substitution profiles of these 10 genes are shown by mapping the sites to the branches of the evolutionary trees. Diverse positive amino acid substitutions were found in these genes (**Figure 4** and **Table 4**). We did observe unequal amino acid substitution profiles among the alleles that arose in 2022. Amino acid profiles of 258S and 258L could both be observed in the MPXVgp010 genes (**Figure 4B**), 543A and 543T could be observed in the MPXVgp098 gene (**Figure 4F**), and 689C, 689H, and 689R could all be observed on the MPXVgp178 of 2022 MPXV isolates (**Figure 4H**).

**Figure 5:**
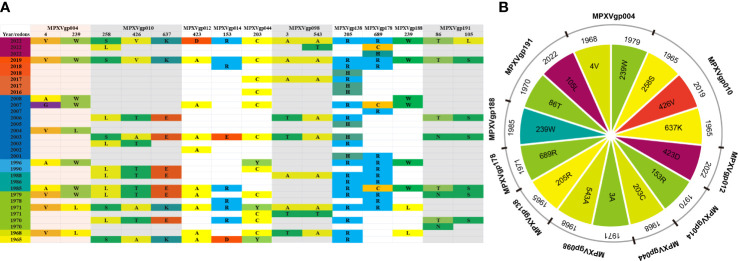
Amino acid substitution profiles of the 10 positive selection genes in a time scale. **(A)** The amino acid sequences at the 10 MPXV genes’ positive selection sites evolved over time. The genes that encode proteins with more than one positive selection sites are highlight with a light color. **(B)** The earliest year when the positive amino acid substitutions arose.

We found that most of the amino acid substitutions (12/15, 80%) occurred several decades before the current outbreak (**Figures 5A, B**). Those alleles with positive amino acid substitutions that could be found in the 2022 outbreak isolates include A4V and L239W of the MPXVgp004 (1968 and 1979), L258S and E637K in the MPXVgp010 (1965), D153R in the MPXVgp014 (1970), Y203C in the MPXVgp044 (1968), T3A and T543A in the MPXVgp098 (1971 and 1968), and H205R in the MPXVgp138 (1965), which occurred about 40 to 50 years before (**Figure 5B**). Only three amino acid substitutions arose recently (in 2019 or 2022), with A423D in MPXVgp012 and S105L in MPXVgp191 appearing in isolates from the 2022 outbreaks (**Figure 5B**), indicating a potential role for further human adaptation of these substitutions for the virus.

### Positive selection mutations have recently caused population growth and are closely related to early virus strains

**Figure 6:**
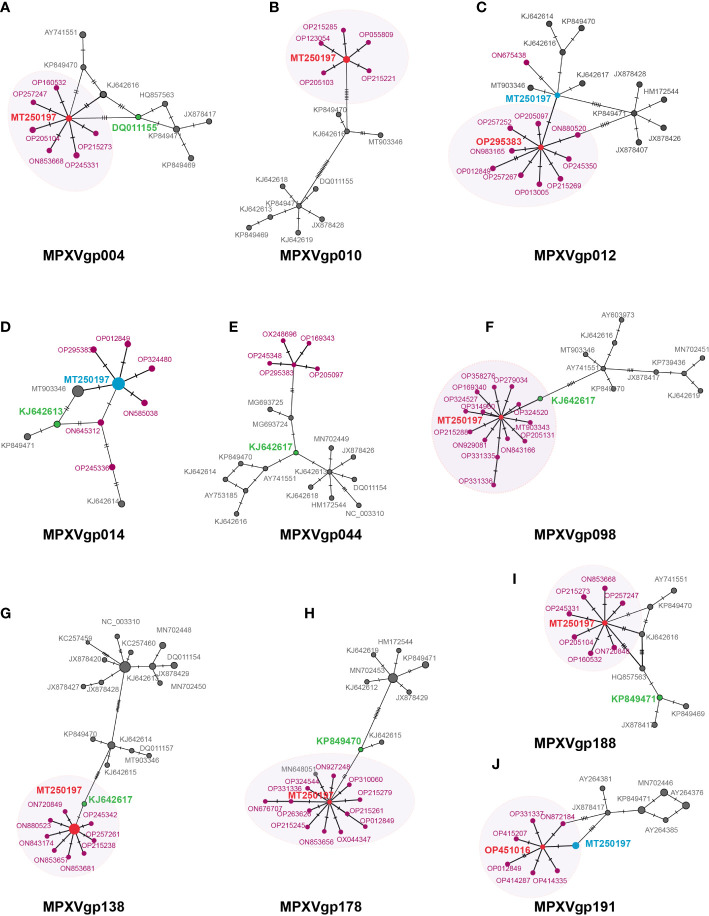
Minimum spanning network of MPXV genes under positive selection at the codon level. Those genes that were identified as positively selected genes including **(A)** MPXVgp004, **(B)** MPXVgp010, **(C)** MPXVgp012, **(D)** MPXVgp014, **(E)** MPXVgp044, **(F)** MPXVgp098, **(G)** MPXVgp138, **(H)** MPXVgp178, **(I)**. MPXVgp188, and **(J)** MPXVgp191 are shown. Haplotype (allele) diversity was obtained from about 1,500 MPXV isolates worldwide. Each oblique line linking between alleles (allele name is shown as its representative isolate NCBI accession numbers) represents one mutational difference. The ancestral allele, or root of the network, is labeled with red, and the represented allele name is marked red. The dark red nodes indicate alleles that arose in the 2022 outbreak isolates. The oval box with a light shadow shows the star structure of the MSNs. For MPXVgp014, MPXVgp138, MPXVgp178, and MPXVgp191, some alleles were identified as the same by popART, which removes the gaps of the alignment, and these alleles show as larger circles. For those alleles, MT250197 is not shown as an obvious center of the star structure and is marked light blue. Those alleles that have positive selection mutations that first arose about 40 to 50 years ago are marked green.

A mutation at a positive selection site should benefit the individuals who bear the mutation. We postulate that mutations in positive selection genes may aid in the spread of MPXV. Some evidence has been obtained from the MSNs of the 10 gene alleles mentioned above (**Figures 6A–J**). Most of the genes with positive selection at the codon level (MPXVgp004, 010, 098, 138,178, and 188) displayed an evident star structure for the alleles of 2022 outbreak isolates with alleles MT250197, which originated in 2019, in the center. MPXVgp012 and MPXVgp191 showed a star structure with alleles from the 2022 outbreak isolates in the center (**Figures 6C, J**). However, only one mutation could be observed among the central alleles (OP295383 of MPXVgp012 and OP451016 of MPXVgp191) and MT250197 in these genes (**Figures 6C, J**). The putative positive selection mutation was found in the central alleles of these MSNs of the eight genes (except for MPXVgp014 and MPXVgp044), indicating a recent population expansion (**Figures 4**, **6**). Furthermore, a close relationship has been observed in MPXVgp004, MPXVgp014, MPXVgp098, MPXVgp138, and MPXVgp178 between the alleles with positive selection mutations in the 2022 outbreak isolates, and those that arose several decades ago (**Figures 6A, D, F, G, H**). There is only one linking step between the ancestral alleles of the 2022 outbreak isolates and those with identical positive selection mutations that arose several decades ago (**Figures 6A, F–H**).

### Positive selection mutations may affect protein functions in a variety of ways

**Figure 7:**
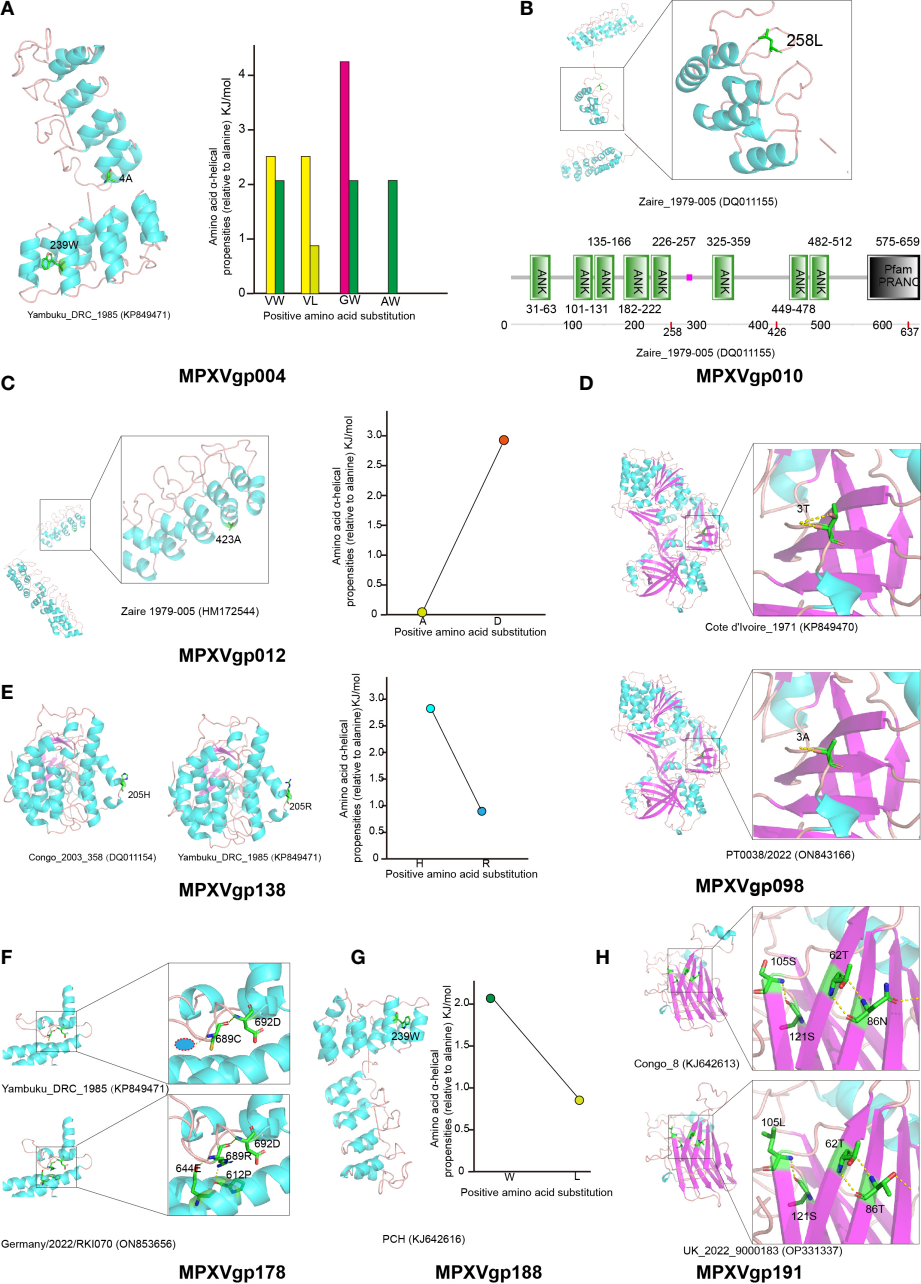
Structure of MPXV proteins that are under positive selection and potential influence of amino acid substitutions in positive selection sites. Secondary structure elements of proteins encoded by positively selected genes are colored in cyan (helix), purple (sheet), and orange (loop), respectively. The amino acid residues that undergo positive selection are shown as sticks. **(A)** Left: The protein structure of MPXVgp004 is shown using strain Yambuku_DRC_1985 as a reference. Right: Amino acid α-helical propensities among different substitution profiles are shown. **(B)** Up: The protein structure of MPXVgp010 is shown using strain Zaire_1979-005 as a reference. Down: Annotation of protein domains and the domain architectures of MPXVgp010. Predictive domains and amino acid residue ranges of each domain are shown. The red dots indicate the location of positive selection sites. **(C)** Left: The protein structure of MPXVgp012 (part) is shown with strain Zaire 1979-005 as a reference. Right: Amino acid α-helical propensities of substitution A423D. **(D)** Up: The protein structure of MPXVgp098 is shown with strain Cote d’Ivoire_1971 as a reference and amino acid residue 3T is shown as a stick. Down: The protein structure of MPXVgp098 is shown using strain PT0033/2022 as a reference and amino acid residue 3A is shown as a stick. Yellow dotted lines indicate H-bonds. The T3A substitution leads to a decrease of H-bond, which may interact with the small subunit of mRNA capping enzyme. **(E)** Left: Protein structures of MPXVgp138 with different amino acid residues at the positive selection sites. Right: Amino acid α-helical propensities among different substitution profiles are shown. **(F)** Comparing MPXVgp178 protein structures with different amino acid residues (689C and 689R) at positive selection sites. The Phyre server could not model MPXVgp178 allele with 689H; thus, it was not shown. Amino acid residues of those that interact with residue 689 are shown as sticks. H-bonds are shown as yellow dotted lines. A red circle wrapped in blue indicates a putative water molecule. **(G)** Left: The protein structure of MPXVgp188 is shown using strain PCH as a reference and amino acid residue 239W is shown as a stick. Right: Amino acid α-helical propensities of substitution (W239L) are shown. **(H)** Comparing MPXVgp191 protein structures with different amino acid residues (86N, 105S and 86T, 105L) at positive selection sites. Amino acid residues of those that interact with residues 86 and 105 are shown as sticks. H-bonds are shown as yellow dotted lines.

The three-dimensional structure of those positive selection genes was modeled using the Phyre server and is shown in **Figures 7A–H**, for predicting the effects of amino acid substitutions on protein function. Positive amino acid substitutions in the ankyrin proteins MPXVgp004, MPXVgp012, and MPXVgp188 all occur in the α-helix of the ankyrin repeat, indicating that amino acid substitution in positive selection sites can significantly change the α-helical propensities (**Figures 7A, C, G**). The amino acid residue alanine at site 4 of the MPXVgp004 is often substituted by glycine, while the residue leucine at site 239 is often substituted by tryptophan (**Figures 4A**, **7A**). All these substitutions may significantly influence the stability of the helical structure of the ankyrin repeat. Similar results could often be observed in the substitution of aspartic acid for alanine at site 423 of MPXVgp012 and the substitution of tryptophan for leucine at site 239 of MPXVgp188 (**Figures 7C, G**). In contrast, we did not find those positive amino acid substitutions in the α-helix structures of MPXVgp010 and MPXVgp178 (**Figures 7B, F**). For MPXVgp010, the Phyre server only yields a part of a three-dimensional structure, where only site 258 could be mapped and located on the loop or linker between the ankyrin repeat (**Figure 7B**). Therefore, we employed SMART (Simple Modular Architecture Research Tool), a web resource (https://smart.embl.de) for the identification and annotation of protein domains and the analysis of protein domain architectures to characterize the positive sites of MPXVgp010 for further study. All three positive sites were not found in the ankyrin repeat but could be located on the loop or linker between the ankyrin repeat (sites 258 and 426). It should be noted that site 637 was included in the PRANC (Pox proteins Repeats of Ankyrin-C terminal) domain (**Figure 7B**), which appears to be related to the F-box domain and may play roles in modulating diverse cellular responses to viral infection by ubiquitin-mediated degradation. Although the MPXVgp178 positive selection site was not discovered in the α-helix of ankyrin repeat, the cystine-to-arginine substitution may significantly increase the stability of α-helix partly formed by 612D and 644E, as additional hydrogen bond (H-bond) could be formed by the C689R substitution (**Figure 7F**).

For those non-ankyrin proteins, including MPXVgp014 and MPXVgp044, positive selection sites could not be mapped to the three-dimensional structure, as the Phyre server only yielded a partial structure not containing those sites (e.g., residues 2 to 141 for MPXVgp014 and 223 to 601 for MPXVgp044). Based on the simple principle that if the amino acid substitutions significantly changed the properties of these residues (e.g., acidic amino acid changed to a basic amino acid) (**Figure 4D**), it could influence the distant residue from the changed position (Montalibet et al., 2006), causing the functional change in the three-dimensional structure. This could explain the functional change of D153R and E153R in the MPXVgp014 (**Figure 4D**). However, the role of the Y203C substitution in MPXVgp044 deserves further research.

We could only obtain part of the three-dimensional structure from the Phyre, which lacks residues 535 to 548 of MPXVgp098 where site 543 is included. One H-bond was reduced after T3A substitution (**Figure 7D**). H205R could be found as a substitution in the positive selection site of MPXVgp138. The sites were discovered to be in an α-helix (**Figure 7E**). The stability of the α-helix containing the substitution may be influenced by H205R, as conformational changes in the α-helix may be caused by the substitution due to arginine’s longer side chain. Unlike the other nine proteins, the mapping of the MPXVgp191 positive selection sites (sites 86 and 205) revealed that they were all located in the β-sheet (**Figure 7H**).

## Discussion

Consisting of ~197 kb with ~190 non-overlapping coding genes, the double-stranded DNA genome of MPXV is responsible for various biological characteristics of the virus and plays a key role in viral host preference/range, host immunomodulation, and viral pathogenicity (Luna et al., 2022). Detailed characterization of the evolution patterns of these coding genes may aid in a thorough understanding of the mechanism underlying the recent emergence of MPXV cases in several regions and outbreaks in multiple countries (Chakraborty et al., 2022). The unusually enhanced human-to-human transmission of hMPX among patients has been discovered in the 2022 outbreak, indicating that the 2022 outbreak isolates have better host adaptation to humans, but how this happens is still unknown. We discovered that MPXV was not a virus whose evolution was frequently driven by intra-species homologous recombination, despite the fact that some poxviruses have high rates of recombination, resulting in the recurrent emergence of tandem gene duplications (Esposito et al., 2006; Sasani et al., 2018), and even this evolution mechanism was proposed in MPXV (Gigante et al., 2022). Only two genes (MPXVgp090 and MPXV182), which functioned as RNA polymerase subunit and surface glycoprotein, were found to have recombinants supported by both the RDP and splits tree.

The ratio of substitution rates, denoted dN/dS, is still a reliable measure to detect proteins undergoing adaptation, which is used to infer the direction and magnitude of natural selection acting on protein-coding genes (Kryazhimskiy and Plotkin, 2008). More than 60% of the MPXV coding genes have an overall average dN/dS < 0.5, indicating that purifying selection is the primary evolutionary direction for MPXV. Eleven of these genes, in particular, were found to have an extremely negative selection (very low dN/dS), implying improved functional stability (Escorcia-Rodríguez et al., 2022), which could also be deduced from the functional annotation of these genes, including DNA- or telomere-binding proteins, virion core proteins, transcription factors, and enzymes (**Supplementary Table 2**). In contrast to these genes favored by extremely purifying selection at the whole gene level, nine MPXV genes, some of which participate in virus–host interaction, including the kelch-like protein (MPXVgp015) (Kochneva et al., 2005), the IL-1 receptor antagonist (MPXVgp016) (Weaver and Isaacs, 2008), the alpha amanatin target protein (MPXVgp024) (Gonzalez and Esteban, 2010), and the caspase-9 (apoptosis) inhibitor (MPXVgp033) (Suraweera et al., 2020), have significantly high dN/dS (>1.5), implying whole gene level positive selection operating these genes or the relaxation of negative selection (Lin et al., 2019). The dN/dS ratio in an amino acid-coding sequence alignment has been extensively used to identify individual codons/sites evolving under positive selection, which could uncover whose signal was “masked” by averaging across the whole sequence, reveal, and speculate functional changes in these sites (Bloom, 2017; Zhan and Zhu, 2018; Zhan et al., 2020). This is particularly important for studying pathogens’ adaptive evolutionary and biological mechanisms for defending against unfavorable environments or promoting host adaptation, transmission, and pathogenicity (Bloom, 2017; Zhan and Zhu, 2018; Velazquez-Salinas et al., 2020; Zhan et al., 2020; Emam et al., 2021). In the present study, 10 MPXV genes were identified as positive selection genes at the codon level and the positive amino acid substitution profiles were uncovered. Most of these genes are involved in virus–host interaction, and some, such as those ankyrin genes (MPXVgp004, MPXVgp010, MPXVgp012, MPXVgp178, and MPXVgp188) and Bcl-2-like genes (MPXVgp014) are likely involved in host range determination (Spehner et al., 1988; Perkus et al., 1990; Opgenorth et al., 1992; Haller et al., 2014). A subsequent study revealed that positive selection was more likely to occur in those host range genes (**Figure 3**), indicating that positive selection in these genes at the given codons may be beneficial to human-to-human transmission in the current outbreak. Aside from genes that determined host ranges, MPXVgp044, which interacts with the host kinesin light chain, wraps the host cell membrane and plays roles in the extracellular enveloped virus (EEV) maturation (Laliberte and Moss, 2010; Carpentier et al., 2015), and MPXVgp138, which is a component of IMV surface tubules, participates in the binding of MVs to the cell surface (Moss, 2016); enzyme MPXVgp098 and one of the host immune modulators, MPXVgp191, were also found to be subjected to positive selection, indicating that substitutions in these genes and the subsequent functional changes may also contribute to the current outbreak (De la Peña et al., 2007).

In terms of genome architecture, MPXV has historically been classified into two major clades (West African and Central African, also known as Congo clades) with mortality rates of <1% and about 10%, respectively (Ladnyj et al., 1972; Simpson et al., 2020). To date, five major hMPX outbreaks have been documented, occurring in 1970, 1996, 2003, 2018, and 2022, respectively (Luna et al., 2022). We discovered that alleles of half of the positively selected genes were clustered into three main clades based on the phylogenetic analysis. Each clade correlates with the different epidemiological hMPX outbreaks, lending support to a new proposal for MPXV classification that divides isolates into three clades (Luna et al., 2022). Despite this, the phylogenetic incongruence among some of these genes suggested possible genetic drift, gene duplication, horizontal gene transfer, or lineage sorting in the evolutionary history of the MPXV population (Jeffroy et al., 2006; Som, 2015). The alleles from the most recent 2022 outbreak isolates were mostly clustered with those originating from the 2018–2019 Nigeria outbreaks, indicating MPXV’s continuous evolution and divergence. However, due to the lower frequency of human-to-human transmission, the majority of the positive selection force is relatively weak, which is consistent with monkeypox as a zoonotic disease with limited human-to-human transmission (Parker et al., 2007). We also discovered that the 2022 isolates still possess alleles that lack putative positive amino acid substitutions in some genes. Site 689 in MPXVgp178 of OP331336 and ON676707, and site 258 in MPXVgp010 of OP0558509, for example, remain unmutated, indicating that a set of MPXV isolates is responsible for the 2022 outbreak, as well as the ongoing selection pressure operating these genes. This emphasizes the significance of tracking the mutation profiles of the MPXV isolates in the current outbreak, which was being done by other researchers (Benvenuto et al., 2022; Gigante et al., 2022; Isidro et al., 2022; Kannan et al., 2022; Wang et al., 2022). While the MSNs of the 10 genes indicate a close relationship between the 2022 outbreak isolates and the 2019 isolate (e.g., MT250197), the starburst pattern with one allele (2019 isolate MT250197 or 2022 isolates OP295383 and OP451016) in the center and many other alleles surrounding the central allele suggests a signature of rapid population expansion and the possibility of the initial effect (Bubac and Spellman, 2016). The discovery of only one step link between the alleles of 2022 isolates and those with positive selection mutations that arose several decades ago further suggests that the more adaptive and circulated alleles are more prevalent in the natural reservoir.

Many amino acid substitutions/mutations occurred among alleles of the 10 genes under codon-level positive selection. Amino acid substitution, for example, occurred in 10 of 437 sites of MPXVgp004, which was five times the number of positive selection sites. Mutations in the amino acid sequence allow proteins to acquire new functions. However, only those mutations at the positive selection sites may significantly improve the survival of or be beneficial to microorganisms with mutated alleles, and thus be fixed. In this study, 15 sites in MPXV’s 10 proteins were subjected to strong positive selection (**Table 4** and **Figures 5A, B**). Some of the three-dimensional structures of those positive selection genes with the positive sites could not be modeled using the Phyre server, because the characterization of poxviral proteins is unfortunately scarce. Most of the positive selection sites are located in the α-helix, which comprises the convex surface “back” of ankyrin proteins’ stacked repeats (sites 4 and 239 for MPXVgp010 and site 239 for MPXVgp188), indicating that the mutation may influence host-range selection (Li et al., 2010). We also found a significant change of amino acid α-helical propensities by the amino acid substitution in the positive selection sites of some genes (MPXVgp010, MPXVgp012, and MPXVgp188), as well as a more H-bond in the MPXVgp178 after a C689R substitution, indicating a potential change in the stability of the ankyrin repeat α-helix in these genes after substitutions (Kumar et al., 2000). Unlike ankyrin proteins, the amino acid substitutions in MPXVgp098 and MPXVgp191 were found in the loop or β-sheet. MPXVgp191 is a chemokine binding protein that moderates host immune response; lacking this gene in the poxviruses may increase disease severity (Townsend et al., 2013; Townsend et al., 2017; Lum et al., 2022), so we supposed that the amino acid substitutions on the two positive selection sites (N86T and S105L) may influence MPXV virulence. As a result, S105L became one of the mutations that drew the attention of the UK Health Security Agency (2022). The MPXVgp098 gene encodes an mRNA capping enzyme subunit that is important in catalyzing the three steps of mRNA capping and regulating virus gene transcription (De la Peña et al., 2007). It is believed that it had no interactions with host proteins (De la Peña et al., 2007). The T543A mutation, according to Benvenuto et al., may stabilize the mRNA capping protein MPXVgp098 (Benvenuto et al., 2022). The T3A mutation, on the other hand, may impair the stabilization of the N-terminal extension to the catalytic site and the precise positioning of the methyl-donor in MPXVgp098 (De la Peña et al., 2007). Because RNA triphosphatase and guanylyl-transferase activities of MPXVgp098 might be influenced by a decrease of one H-bond when T3A substitution occurs, we supposed that the positive amino acid substitution may have an effect on transcription initiation in the host cells (Grimm et al., 2021). However, further biological experiments are required to validate our hypothesis.

## Conclusions

Incorporating intensive molecular evolution research, we discovered that intra-species homologous recombination occurred rarely among the MPXV genes, whereas 10 genes that were mostly associated with virus–host interaction, particularly those that determine the virus’s host range, the ankyrin genes, and a Bcl-2-like gene, were found to have codon-level positive selection, which may aid the virus’s adaptation to humans in the context of the 2022 outbreak. Positive selection amino acid substitution profiles of the 10 genes were uncovered and mapped to these genes’ phylogenetic trees. Interestingly, the majority of the positive mutations (12 of 15, 80%) were discovered in the virus isolates from several decades ago, but spread across almost all alleles of the 2022 outbreak isolates. The 2022 outbreak alleles are closely related to those that emerged several decades ago and have since experienced population expansion, implying that these genes have been continuously adapted and that a reservoir of the more adaptation alleles has circulated in its natural reservoir (e.g., some susceptible animals). The three or, more precisely, the two never-before-seen positive mutations (A423D in MPXVgp012 and S105L in MPXVgp191) may play a special role for MPXV, allowing it to spread more easily among people. hMPX has already accelerated human-to-human transmission. Monitoring the gene mutational landscape, particularly identifying positive selection mutations after the virus’s widespread transmission among humans, is crucial for predicting changes in virulence and transmissibility. Our research on the molecular evolution of MPXV at a single-gene level will fill some knowledge gaps about the virus and provide clues for the unusual emergence of the current outbreak.

## Data availability statement

The datasets presented in this study can be found in online repositories. The names of the repository/repositories and accession number(s) can be found in the article/**Supplementary Material**.

## Author contributions

X-YZ and YH conceived and designed the study. X-YZ and GZ analyzed the data. YH verified the data. X-YZ made the data interpretation. X-YZ wrote the manuscript. X-YZ revised the manuscript. All authors contributed to the article and approved the submitted version.
