# Supplementary material for: Evolutionary dissection of monkeypox virus: Positive Darwinian selection drives the adaptation of virus–host interaction proteins
Source: Front Cell Infect Microbiol. 2023 Jan 13;12:1083234. doi: 10.3389/fcimb.2022.1083234 (PMC9880225; doi:10.3389/fcimb.2022.1083234)
Supplement: Supplementary file 3 [file Table_2.docx]

**Supplementary Table 2. Details of MPXV genes undergo whole gene level extremely negative or positive selection.**

| **Gene product** | **Gene name** | **Gene name based on Vaccinia virus nomination** | **Gene sequence length (bp)** | ω | **Function based on NCBI annotation** |
| --- | --- | --- | --- | --- | --- |
| **Genes undergo extremely negative selection at the whole gene level** | | | | | |
| MPXVgp036 | C10L | F4L | 960 | 0.0001 | Ribonucleotide reductase small subunit R2 |
| MPXVgp050 | F1L | E1L | 1440 | 0.09921 | Poly (A) polymerase catalytic subunit (VP55) |
| MPXVgp062 | I1L | I1L | 938 | 0.09412 | DNA-binding core protein |
| MPXVgp067 | I6L | I6L | 1149 | 0.09623 | Telomere-binding protein |
| MPXVgp071 | G2L | G3L | 336 | 0.00010 | Entry/fusion complex component |
| MPXVgp099 | E2L | D2L | 441 | 0.06208 | Virion core protein |
| MPXVgp111 | A1L | A1L | 453 | 0.08878 | Late gene transcription factor, VLTF-2 |
| MPXVgp114 | A4L | A3L | 1935 | 0.07854 | Major virion core protein p4b |
| MPXVgp119 | A9R | A8R | 879 | 0.08664 | Intermediate transcription factor, VITF-3, 34 kDa |
| MPXVgp123 | A13L | A12L | 573 | 0.06980 | Virion core and cleavage processing protein |
| MPXVgp164 | B3R | B1R | 912 | 0.09143 | Ser/Thr Kinase |
| **Genes undergo extremely positive selection at the whole gene level** | | | | | |
| MPXVgp015 | D12L | C5L | 621 | 2.77453 | Kelch-like protein |
| MPXVgp016 | D13L | C4L | 951 | 2.75675 | IL-1 receptor antagonist |
| MPXVgp024 | P2L | N2L | 534 | 1.63134 | Alpha amanatin target protein |
| MPXVgp030 | C4L | K4L | 1275 | 1.909 | Phospholipase-D-like protein |
| MPXVgp033 | C7L | F1L | 1108 | 1.55019 | Caspase-9 (apoptosis) inhibitor (mitochondrial- associated) |
| MPXVgp131 | A21L | A21L | 348 | 2.77927 | IMV membrane protein, entry/fusion complex |
| MPXVgp133 | A23R | A22R | 564 | 2.91103 | Holliday junction resolvase |
| MPXVgp156 | A46R | A45R | 378 | 2.77925 | Inactive Cu-Zn superoxide dismutase-like virion protein |
| MPXVgp182 | B21R | N/A | 5643 | 1.77937 | Surface glycoprotein |

N/A indicates not available.
