# Supplementary material for: Evolutionary dissection of monkeypox virus: Positive Darwinian selection drives the adaptation of virus–host interaction proteins
Source: Front Cell Infect Microbiol. 2023 Jan 13;12:1083234. doi: 10.3389/fcimb.2022.1083234 (PMC9880225; doi:10.3389/fcimb.2022.1083234)
Supplement: Supplementary file 5 [file Table_4.docx]

**Supplementary Table 4. Details of ankyrin genes in MPXV.**

| **Gene product** | **Gene name** | **Gene name based on Vaccinia virus nomination** | **Gene length (bp)** | **LRT P value (M0 Vs. M3)** | **LRT P value (M1a Vs. M2a)** | **LRT P value (M7 Vs. M8)** | **Average ω of the whole gene** | **Function based on NCBI annotation** |
| --- | --- | --- | --- | --- | --- | --- | --- | --- |
| MPXVgp003 | J3L | N/A | 1767 | 0.999999693 | 0.999361204 | 0.998976524 | 0.54460 | Ankyrin |
| **MPXVgp004** | **D1L** | **N/A** | **1314** | **0.053119982** | **0.027500475** | **0.020266926** | **0.74079** | **Ankyrin/Host Range** |
| **MPXVgp010** | **D7L** | **D8L** | **1983** | **0.034038793** | **0.010929672** | **0.010895472** | **0.96227** | **Ankyrin/Host Range** |
| **MPXVgp012** | **D9L** | **C9L** | **1893** | **0.028525305** | **0.008523263** | **0.144727155** | **0.76806** | **Ankyrin; Type I IFN resistance** |
| MPXVgp025 | O1L | M1L | 1341 | 0.999999819 | 0.999469141 | 0.999629069 | 0.72143 | ANK-containing protein; apoptosis inihibitor |
| MPXVgp027 | C1L | K1L | 855 | 1.000000000 | 0.999743033 | 1.000000000 | 0.4935 | Ankyrin/NFkB inhibitor, host range |
| MPXVgp166 | B5R | B4R | 1686 | 0.403074886 | 0.441098091 | 0.355898354 | 0.50759 | Ankyrin |
| MPXVgp168 | B7R | B6R | 531 | 0.311165715 | 0.112294657 | 0.105348961 | 1.15432 | Ankyrin-like protein |
| **MPXVgp178** | **B17R** | **B20R** | **2364** | **0.03910529** | **0.017571535** | **0.073626446** | **0.6559** | **Ankyrin** |
| **MPXVgp188** | **N4R** | **N/A** | **1314** | **0.120056555** | **0.038311468** | **0.244555745** | **0.74175** | **Ankyrin** |
| MPXVgp189 | J1R | C19L | 1767 | 0.999999742 | 0.999170344 | 0.999036464 | 0.65072 | Ankyrin |

Bold indicates that these genes were identified as positive selection genes at the codon level, and the red indicates those parameters elucidate adaptation signatures within the gene.
